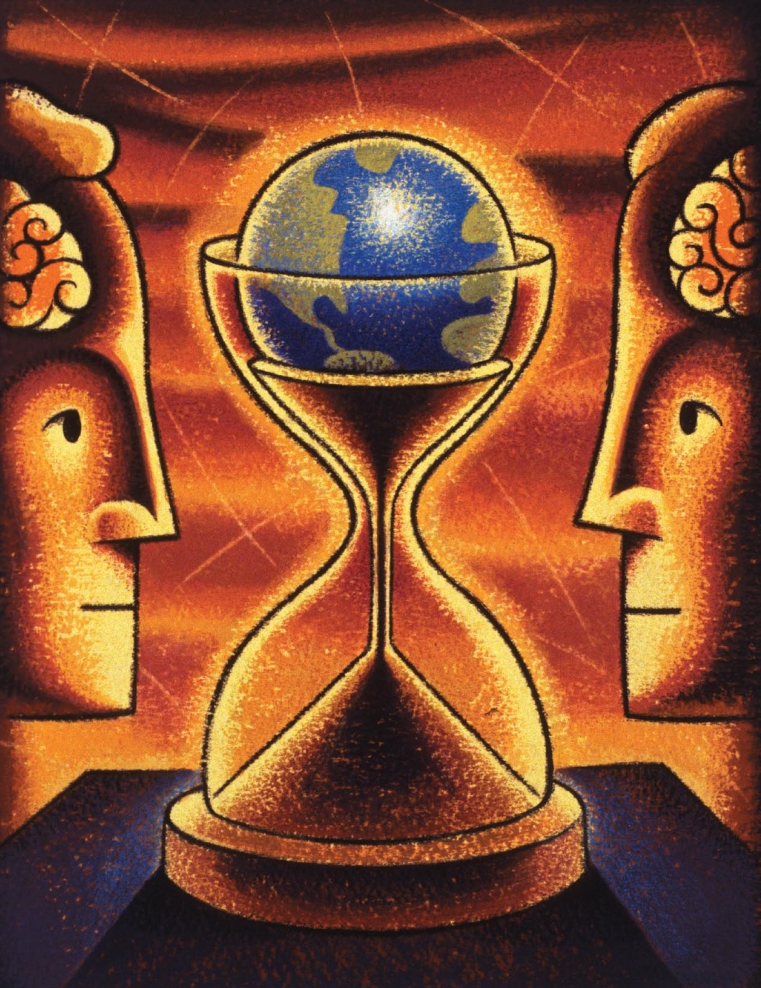# Beyond Mitigation: Planning for Climate Change Adaptation

**DOI:** 10.1289/ehp.117-a306

**Published:** 2009-07

**Authors:** Charles W. Schmidt

**Affiliations:** **Charles W. Schmidt**, MS, of Portland, Maine, has written for *Discover Magazine, Science, and Nature Medicine*. In 2002 he won the National Association of Science Writers’ Science-in-Society Journalism Award

Consider the floods, plagues, famines, and other calamities we can expect from climate change, and an apocalyptic prophecy might come to mind, perhaps rightfully so. An expert panel convened to assess risks from climate change put it this way in the 16 May 2009 issue of *The Lancet*: Should global mean temperatures rise an additional 5–6°C, “more than a billion people could be dispersed in environmental mass migration. . . . An additional 2 billion would be water stressed while billions more would face hunger or starvation. The risk of armed conflict would rise. Public health systems around the world would be damaged, some to the point of collapse.”

Alarming scenarios like this have fueled efforts to lower heat-trapping greenhouse gas emissions and limit future impacts [see “Climate Change Abatement Strategies: Which Way Is the Wind Blowing?” p. A296 this issue]. But more recently, scientists have acknowledged that some degree of global warming is now inevitable. “Climate change models tell us that even if we blocked all emissions now, the amounts of greenhouse gases already in the atmosphere would raise global temperatures by an additional 2°C by 2100,” says Robert Corell, vice president of the John Heinz III Center for Science, Economics, and Environment, in Washington, DC. In light of this probability, Corell says, mitigation has begun sharing the global policy stage with a new challenge: how to adapt to climate change that is already under way.

## Health Effects: More of the Same?

Adaptation refers to the measures humans can take to minimize damage from climate change—for instance, by protecting infrastructure and communities against flooding, erosion, and extreme weather, or by preparing for changes in precipitation that result in too little—or too much—rain to sustain traditional crops.

Adapting to climate change’s anticipated health problems is a more recent concern, says Kristie Ebi, a Virginia-based independent consultant on health issues related to climate change. U.S. officials in particular have been slow to consider health adaptation, she says, both because of funding shortages in this area and because climate-related health problems in this country aren’t yet as acute as they are elsewhere in the world.

Other regions have not been as fortunate. In Europe, for instance, an unprecedented heat wave in the summer of 2003 killed more than 52,000 people and contributed to heightened wildfire activity and crop failure. Sustained high temperatures across the northern portion of the continent surprised and confounded countries accustomed to milder summers.

Health problems attributed to climate change are expected to increase worldwide in the coming decades, Ebi says, not just as a downstream consequence of flooding, drought, forest fires, and other area-specific outcomes of climate change, but also from increasing heat stress, the spread of vector-borne pathogens into new territory, and increased exposure to allergens and air pollutants such as ozone, which is formed by heat-driven reactions involving smog and sunlight.

Climate change won’t unleash new health effects so much as it will intensify existing problems, says Jonathan Patz, an associate professor of environmental health at the University of Wisconsin–Madison. “[A warming climate] isn’t going to dramatically alter the types of illnesses we’re dealing with. We’re dealing with them today, and global warming will affect them all.”

Because climate change will affect different regions in different ways, adaptation is chiefly a local concern. But adaptation poses difficult challenges, given the paucity of information on projected impacts at local levels. The global models that scientists use to predict air temperature, precipitation, and other climate variables can’t yet achieve spatial resolutions better than 150–350 km, according to Cynthia Rosenzweig, director of the Climate Impacts Group at the Goddard Institute for Space Studies and an advisor to New York City’s adaptation program. However, mountain ranges and other topographic features can influence the local atmosphere at much finer scales, leaving decision makers unsure what to expect in specific areas.

Given data shortages, local health impacts are hard to predict, Ebi says. “When I speak at congressional hearings on Capitol Hill, committee members invariably ask me what sorts of health effects they can expect in their own districts,” she says. “And I tell them, ‘Without the detailed studies that we need, I just don’t have the data to tell you.’ We have some detailed projections of how ozone might change in New York and some limited projections in California, but that’s it. The same applies to allergens and diarrheal diseases from food- and waterborne pathogens, which are sensitive to changes in temperature and precipitation. How can cities prepare for adaptation when they can’t estimate the size of the impact?”

## Action at the Local Level

In the June 2009 issue of *EHP*, Ebi and colleagues concluded that federal extramural funding for research directly studying the human health effects of climate change was less than $3 million annually, an amount they called “inadequate to address the real risks that climate change poses for U.S. populations.”

Now, with communities and states asking the federal government for help with climate change adaptation planning, Ebi says, the federal government’s involvement in adaptation appears to be growing. The Centers for Disease Control and Prevention (CDC), for instance, through the National Center for Environmental Health, has been coordinating workshops for local, state, and city health departments with the aim of helping these entities understand potential health effects related to climate change, especially among the most vulnerable populations, and how to address them. The CDC also received $7.5 million in new funding to study climate change and public health for fiscal year 2009.

The U.S. Environmental Protection Agency (EPA) Climate Change Science Program has investigated a number of adaptation strategies targeting human health. They include more surveillance and training to address the spread of vectorborne diseases such as malaria and dengue fever. They also include optimal land use designs—such as more use of undeveloped “green space” and shade trees—to help keep city-dwellers cool as well as better weather advisories to warn of acute temperature extremes. According to the EPA, government officials can plan for alternative water supplies and engineers can protect aquifers from saltwater intrusion. Coastal dikes can protect against rising sea levels and storm surges. And in some cases, populations and vulnerable infrastructure can be relocated away from threatened coastlines altogether.

A key point, says Patz, is that mitigation and adaptation strategies can have mutual health benefits. As an example, he points to a shift toward replacing pavement with green space, which he asserts can cut energy use and help lower local temperatures in the summer. City dwellers live in “urban heat islands,” Patz explains, where black asphalt in parking lots and roads can render summer heat virtually intolerable. “Transportation accounts for one-third of the world’s oil consumption,” Patz says. “So, by building shaded bike and walking trails, we have a great opportunity to make ourselves less dependent on cars while also reducing the number of Americans who don’t get recommended levels of exercise.”

In California’s *Public Health Climate Change Adaptation Strategy*, currently in draft form, officials wrote that they will “promote community resilience and reduce vulnerability to climate change” chiefly by “altering the built environment with more urban residential density, bike trails, and parks designed to lessen effects from urban heat islands.” Other measures described in the strategy include expanding community and school gardens, educating health care providers about climate change, conducting health assessments of proposed mitigation and adaptation responses, and strengthening local emergency response for climate impacts.

As for whether any of these strategic elements are being funded, Linda Rudolph, deputy director in the California Department of Public Health, says, “We’re in the same boat as any other state struggling with budget woes, doing the best we can within existing programs. That said, when you look at the breadth and scope of the likely impact of climate change, you can’t escape from the broad nature of the threat. Climate change threatens our food, water, shelter, and the basic components on which we depend for survival.”

California is one of dozens of states, counties, and cities in the United States, most of them coastal, that have been drafting their own adaptation strategies. Another is New York City, where Mayor Michael Bloomberg has taken a lead role in this area. Rosenzweig says roughly 40 agencies—both public and private—are involved in the New York City adaptation effort. An important activity, Rosenzweig says, has been to estimate the city’s vulnerability to sea-level rise, which is potentially the most damaging climate-related change facing New York.

To project the local rise in sea level, Rosenzweig and her colleagues calculated two scenarios: one that does not account for rapid ice melting in polar regions and one that does. With the former analysis, she says, New York City faces a 2-foot sea-level rise by 2080. In the latter, that figure doubles to 4 feet over the same duration, enough to threaten some of the city’s most crucial infrastructure, including JFK and La Guardia airports, which were built on drained coastal wetlands. Asked how these scenario depictions could guide future policies, Rosenzweig says, “Engineering designs in New York were built to accommodate a hundred-year flood. But with sea-level rise, we’re going to have to rethink this. We’ve found that low-lying areas of the city face an ever-increasing risk of coastal flooding. ”

Rosenzweig concedes that model projections aren’t perfect. Scientists have generally relied on global climate model simulations, she explains, which have a longer history of use and provide more robust predictions than those generated by regional models developed chiefly for studies of weather on shorter time frames. New York City’s scenarios were derived by linking global and regional models using evolving techniques to align the different models’ parameters.

“Regional climate models have resolutions of fifteen to fifty kilometers, but these are used mainly in a research mode,” Rosenzweig says. “As regional climate modeling and computer power improves, the more detailed information can be incorporated into decision making. But the key lesson is: Don’t wait for the perfect model! There is ample information coming from the global climate models to begin to plan regional adaptation strategies now. Adaptation is a process, and it’s going to evolve through time.”

## Looking for Decision Support

Along those lines, adaptation across the board relies on “the tools of day-to-day governance,” says Lara Whitely Binder, an outreach specialist with the University of Washington’s Climate Impacts Group, which studies climate change in the Pacific Northwest. This is unlike mitigation, she explains, which is more generally concerned with new energy infrastructure and technology. “Adaptation is about using fee setting, conservation planning, bond issuances, infrastructure upgrades, and other things that are already in the governance toolbox,” she says. “But it’s also about using them in a way that’s appropriate for climate change.”

What local policy makers addressing adaptation need, the National Research Council recently concluded, is information to make sound decisions. In a report released 12 March 2009, *Informing Decisions in a Changing Climate*, National Research Council panelists proposed that federal leadership is essential in this area. One initiative highlighted by the National Research Council is the Regional Integrated Sciences and Assessments (RISA) program developed by the National Oceanic and Atmospheric Administration, through which university-based scientists help decision makers evaluate local impacts and response options. The University of Washington Climate Impacts Group is one of nine RISA programs now in existence.

Corell likens the RISA programs to the cooperative extension system. “We put cooperative extensions between the scientist and the farmer so that one can learn from the other,” he says. “Cooperative extensions aren’t run by any one agency, and similarly many agencies can provide decision support for adaptation depending on the expertise that’s needed.”

On the global front, Bo Lim, a technical advisor on climate change adaptation with the United Nations Development Programme (UNDP), says that program’s adaptation budget jumped from $15 million in 2006 to $200 million in 2009. Lim says many developing countries are particularly hard-pressed to adapt because of their poverty and geographic vulnerability to the effects of climate change (for instance, increased storm activity and even relatively small rises in sea level can be particularly problematic for small island nations). These countries will no longer negotiate on mitigation without more financing for adaptation from wealthier countries, she says.

Most of the UNDP’s adaptation resources are going to Africa, the small island nations, and several countries in Latin America. But Lim points out that the allocations aren’t equitably distributed chiefly because funding decisions aren’t based on indices of vulnerability or human development. Because countries have been unable to agree on such frameworks, the money tends to go to countries that know how to work the system, she admits, “and the fastest ones get the money first.” What’s more, of the $200 million allocated annually to adaptation, only $10 million goes exclusively to health programs to combat threats such as heat stress and vectorborne disease transmission, she adds. The rest goes for technical assistance in food security and agricultural production.

To access adaptation funding, countries have to show that the primary driver for the problem is climate change and not underlying societal phenomena such as failing health systems. “We’re really looking for technical evidence of climate impacts,” Lim explains.

The crucial need, Lim adds, is that adaptation occur within a framework for sustainable development. “For us, adaptation has to be aligned with UNDP’s corporate mission, which is to alleviate poverty among the poorest one billion people on the planet,” she says. “So, from our view, we’re looking at how we can create, support, and build national institutions that can respond to climate change. You need decentralization and clear budgeting, which are really bread-and-butter practices for good governance.”

Adaptation to environmental stressors is not a new concept, but it’s taken on a new shade of meaning as climate change awareness has grown. “[We’re going to have] climate impacts for many decades to come,” says Lim. “This means that adaptation today is no longer tomorrow’s choice, but today’s imperative.”

## Figures and Tables

**Figure:**